# Annotations

**Published:** 1888-02-25

**Authors:** 


### ANNOTATIONS.

**Wicked or Weak?**

A GENTLEMAN applied at one of the metropolitan police-courts last week to ask the magistrate's advice in a case of a nature by no means uncommon. He stated that his son, a lad of nearly sixteen, was entirely beyond his control, and as a last resource he had determined to send him away. The boy had been brought up at an expensive school, but had not been able to get beyond writing and ciphering. In reply to questions from the magistrate, the father said the boy was in the habit of lying and staying out at nights. He had been treated kindly and reasoned with, and had not been thrashed for nine months. Presumably, therefore, the persuasion of the rod had been tried as well as other means. The father being in good circumstances the boy was not eligible for an industrial school, and Mr. Partridge was obliged to confess that he could not help in any way. Every experienced medical man is familiar with cases of this kind, and is often much troubled to know what to do with them. Only last week a similar case, of a boy of seven, was brought to the writer, the mother stating that her friends urged upon her the duty of taking him to a "mad" physician. Doctors can do nothing for such patients except this—and it is very important—they can explain to the parents that they are cases of defective development, and that what is wanted is not physic, and certainly not punishment, but teaching. These unhappy children cannot be called idiots, but they are only a little less deficient than idiots, and should be treated on the same lines. As a rule, office or any other merely brain work is the last calling they should be put to. The elements of education should be given to them, and along with such teaching they should have plenty of play, and be taught some healthy occupation, such as market gardening, farming, carpentering, etc. Many who are now permitted to grow up without the training best fitted for them sink down from bad to worse when they are released from parental control, and often bring both themselves and their families into public disgrace. The blame to them is very little; the pity they deserve is very great. An institution for children of this kind belonging to the better classes would be invaluable. How far behind, as a community, we are in real intelligence and capacity could hardly be more convincingly shown than it is by the fact that such an institution has yet to be founded.

**Cock-Fighting.**

Do any of our readers know anything of the noble and inspiring pastime of cock-fighting? It occurred to the writer some years ago to be called from the breakfast-table on a cold morning in winter, and informed that a "main of cocks" was to take place not far from the village where he was then residing. He sent a note to the superintendent of police at the nearest town, and proceeded on horseback to the spot where the "main" was to be fought. A "main of cocks," it should be explained, consists of several pairs which are fought in succession. The place of that particular event was an old disused stone quarry, which was filled with a crowd of some four or five hundred men—the very pick and essence of the ruffianism of a large colliery district. The game was in full swing. Two gamecocks were fighting each other in most approved fashion, with two brave and generous human beings to hand them. The others made a ring and looked on with every manifestation of fiendish delight. When the writer rode into their midst he was assailed with the choicest epithets the "Scoundrels' Dictionary" could supply. He was bidden by a hundred tongues at once to "go to H——," and told in peremptory tones that if he did not "clear out" forthwith he would be sent there before he was five minutes older, and stones began to be thrown from the outside of the crowd. Being one among four or five hundred, and in a lonely place, he thought it better to slowly beat a retreat in the direction whence he hoped to meet the approaching police; who, as is not unusual, never appeared until the "main" was over and the savage crowd had dispersed. He saw enough, however, to convince him that any man who deliberately encourages and takes part in cockfighting is a ruffian for whom the only proper reward is the lash. Two cocks, with their wings clipped, and the feathers plucked until they were nearly naked, with sharp-pointed steel "spurs" two inches long placed "sheathwise" upon the spurs of each—rushing at one another like demons, striking with the deadly steel about head, eyes, and body, until one or other fell covered with blood, and exhausted or killed—such was the sight. This manly sport is now being extensively revived, and several times since the New Year came in the daily papers have reported fights in different parts of the country, but especially in the south. At those fights members of the aristocratic and professional classes are said to have been present, as well as wretches of baser degree. To such manners has the age of Mr. Matthew Arnold and Mr. Herbert Spencer come! For violent thieves, wife-beaters, and cockfighters the lash should be put freely into requisition, until their fiendish cruelty is whipped out of them or they are proved fit only for permanent felon's life.

**Early Marriages.**

THE Archbishop of Canterbury has done well to call public attention to the stupid folly and the serious evil of early marriages. His Grace said that in his part of London it almost made his blood run cold to think of them. There was an enormous number of minors who did not think it any harm, with no means in the world, to set up and begin "to bring a family into the world." From a medical point of view very early marriages are absolutely indefensible, and ought to be legally prohibited. Medical men, unfortunately for the State, have never taken any considerable part in public affairs, and hence among practical politicians they are not considered of much account. But this question is emphatically one for physiologists, and it cannot be doubted that their condemnation would be stern and unanimous. There is hardly a word to be said in favour of very early marriages, even from the moralist's point of view, whilst from the scientist's the reasons against them are numerous and overwhelming. They are first of all highly injurious to the boys and girls themselves, plunging them into the midst of cares, anxieties, and responsibilities for which they are in no way fitted. If children be born to them, they are, according to the little understood but constantly operative laws of heredity, physically and mentally immature, like their immature parents, and seriously disqualified for growth and development in unfavourable circumstances. They are, moreover, likely to grow up, according to the same law, reckless and undisciplined, like their reckless and undisciplined progenitors. With the weakening of the physique the mental and moral forces are weakened, and the third generation of such creatures may be practically incapable of any of the higher duties of manhood. If such marriages were to become general in a community, that community would speedily cease to be able to maintain an independent existence. As it is, the children of these early marriages among workmen often become profligates, thieves, and law-breakers of all kinds; and in cases where they do not descend to those low levels they are the helpless slaves of their robuster neighbours, and after leading a life of incapacity and semi-starvation, often end their days in the poorhouse, if not in the prison. If medical men were as courageously patriotic as they are scientifically intelligent, there would speedily be an effort made to deal with these mad marriages by Parliamentary statute. It is much to be deplored that the members of the medical profession avoid politics, as likely to injure their professional position. Such unmanly timidity is in the highest degree reprehensible.

